# Disease and Sickness

**Published:** 1890-03

**Authors:** 


DISEASE AND SICKNESS, by Samuel Swan, M. D. New York. Reprinted from *The Medical Current*, October, November, and December, 1889.

This pamphlet of six pages is devoted to two objects—one to prove the well-known maxim of the author that "morbific matter will cure the disease which produced it, if given in the highest potency and to any other person than the one from whom it was obtained."

The other object is the advocating of a *mixture* of remedies for any sick condition which exhibits symptoms which cannot be covered by a single remedy. He proposes to take the twelve tissue remedies and potentize them together to the five millionth potency. This combination then is to be administered, like a single drug.

He argues in favor of it by pointing to Edison's success in causing a dozen separate electrical currents to pass along the same wire without interference with each other.

